# The varicocele argument resurfaces

**DOI:** 10.1007/s10815-018-1160-2

**Published:** 2018-03-27

**Authors:** Sherman Silber

**Affiliations:** Infertility Center of St. Louis, St. Luke’s Hospital, Saint Louis, MO USA

**Keywords:** Varicocele, Male infertility, Regression toward the mean

## Abstract

A recent series of articles and reviews published in Fertility and Sterility have rekindled the more than half century debate on varicocelectomy. Every one of these articles favored strongly the repair of varicocele for male infertility. Since my review paper on this issue in 2001, published in Human Reproduction Update, and since advent of ICSI in 1993, I had thought that most reproductive physicians felt negatively about the benefit of varicocelectomy. However, more recent urological papers are causing this negative view to be re-evaluated. It is now advocated by some urologists that varicocelectomy improves sperm count and testosterone levels, and even improves the results with ICSI. Thus, it may be appropriate to revisit older studies again and review the newer ones in this never ending controversy. Newer studies are re-opening the door to review and possibly re-instate varicocelectomy. This dilemma may never be fully resolved, but it is important to keep an open mind.

There is probably no subject that has been more controversial in the area of male infertility than varicocele. Most non-urologist infertility specialists in the world are skeptical of the role of varicocele or varicocelectomy in the treatment of male infertility, especially since the advent of ICSI [1]. However, many urologists strongly recommend varicocelectomy, and now in 2017, there is another push to re-establish this surgery for infertile couples with male factor infertility. The September 2017 issue of fertility and sterility was dedicated to varicocelectomy with all positive, and no negative, reviews [2]. It has been suggested that in addition to infertility, varicocele has a negative impact on Leydig cell function, testosterone level, and overall “male health,” and that varicocelectomy will raise testosterone levels and improve “overall male health” [3–5]. It has also been suggested that varicocelectomy improves the results of ART (assisted reproductive technology) even though most early papers on ICSI showed no relation of sperm parameters to success or failure [6–9]. However, another equally large and very similar cohort study showed no difference in success with ICSI in men with varicocele who underwent varicocelectomy than in men who did not [10]. Interestingly, the study which showed improvement was only when there were younger female partners and not older female partners. To confuse things more, a meta-analysis of these studies concluded no improvement in pregnancy rate with varicocelectomy performed before ART, but there was improvement in birth rate [6]. With azoospermia TESE cases, there was only a “strong trend” toward improvement (but not statistically significant) after varicocelectomy [6]. Because of this enthusiastic resurgence of interest in varicocelectomy, I would like to once again review previous literature to objectively evaluate whether it is appropriate to perform varicocelectomy for male infertility in the era of ICSI.

There have been several, credible, studies in the past which show no effect of varicocelectomy on fertility [11–14]. However, newer studies are showing an effect on varicocele. There were also in the past a few “controlled” studies that favor varicocelectomy, but they have been disregarded because of patient selection bias and protocol deviations [10, 11]. The weight of evidence in the past has been against varicocelectomy. For example, reports that semen parameters are improved by varicocelectomy were troubled by uncontrolled observations and the failure to take into account the extreme variability of semen analysis and its regression toward the mean [15–20]. Many controlled studies have demonstrated that, because of this variability, men with an initially low sperm count tend later to have higher sperm counts in the absence of any treatment whatsoever, because of regression toward the mean. Randomized control trials (RCT’s) should eliminate this effect on sperm count of regression toward the mean. But these RCT’s are very scarce, often possibly because urologists who have this interest might naturally be biased. Therefore, it will be useful to re-evaluate whether past studies on varicocele, sperm count, and fertility might have been flawed or not.

It is commonly known that a left varicocele occurs in 15–20% of all men. In fact we found a large varicocele in 17% of fertile men coming for vasectomy reversals who clearly were fertile before their vasectomy [21]. It has been claimed, however, that in infertile men, 35% or more have a varicocele [2, 22]. However, the incidence of an easily discoverable and palpable, visible varicocele is in fact similar (15–20%) with infertile and fertile men [23, 24]. The problem with such claims of high varicocele incidence is that for infertile males, (or more precisely the male partner of an infertile couple alleged to have some abnormality in semen analysis parameters), there is an intense effort by the examining urologist in such cases to find a varicocele. So for infertile males, there is a bias toward finding a varicocele that does not exist in a routine normal population physical exam. In fact, for infertile men, there has been an intense hunt for varicocele ever since the astonishing case report of Tulloch in 1955 alleging that an azoospermic man developed a high sperm count and fertility after varicocelectomy [25].

Varicocelectomy is not without complications, such as hydrocele or even testicular atrophy. Microsurgery for varicocele, which I originally introduced, has reduced these complications dramatically. Nonetheless, these occasional complications of varicocelectomy have been known for > 20 years. Of course, a microsurgical approach to varicocelectomy should avoid such complications [26–28]. Nonetheless, the occasional serious risk of varicocelectomy cannot be disregarded. The more common risk for post-operative hydrocele (5%) is obviously just a nuisance, and not as serious as devascularization.

Semen analyses are often highly variable, and spontaneous pregnancies without treatment are so common that there is much skepticism about many treatments for male infertility [12, 15–20, 29–35]. No treatment of male infertility is without the risk of delaying more effective treatment until the wife is older. So we should be cognizant of the pitfalls of trying to evaluate either pregnancy results or sperm count results in patients undergoing varicocelectomy. The same critique applies even to recent papers suggesting that varicocelectomy can reduce the need for ART [36, 37].

In 1989, we reported a 10-year follow-up of men undergoing vasovasostomy (who had spermatozoa in the vas fluid without secondary epididymal blowouts) and their long-term results [21]. Even many years later, their fertility was not affected by the varicocele. A decade later, essentially the same question was addressed when varicocelectomy was performed simultaneously with vasovasostomy in vasectomy reversal patients who had varicocele, but varicocelectomy was not carried out in the other vasectomy reversal patients who had a varicocele. There was no statistically significant difference between the two groups.

## Evidence-based practice of medicine

In 1995, Nieschlag proposed a basic axiom that needs to be followed in male infertility treatment: “Therapeutic interventions in male infertility should be based on properly controlled clinical trials” [13]. Several reports on spontaneous pregnancy rates with no treatment in couples with severe male factor justify Nieschlag’s axiom. In 1993, Hargreave reported on patients with severe oligozoospermia, high serum FSH concentrations, and varicocele whose wives became pregnant after an initial infertility consultation without any treatment of the male [14]. A total of 33% of men in this category had a varicocele and did not have time to undergo varicocelectomy before their wife became pregnant. The point of his study was that, with alarmingly low sperm counts, fertile women can become pregnant without any treatment of the male, verifying concepts that have been clear for many years [32–35].

To understand the importance of a controlled study in evaluating the validity of varicocelectomy, one has only to look at the spontaneous conception rates in the wives of men with various low sperm counts. Baker and Burger in 1986, reported life-table pregnancy rates over 3 years in couples with varying categories of semen parameters compared to control groups [19]. Although low sperm counts resulted in lower pregnancy rates, a substantial percentage of couples achieved pregnancy spontaneously despite severe oligoasthenozoospermia [38]. In 1983, Schoysman reported an extensive 12-year experience following 1291 oligozoospermic men who underwent no improvement in semen parameters [35]. These studies demonstrated the difficulty of interpreting whether any treatment of the male with oligozoospermia, e.g., varicocelectomy, has any discernible effect on the pregnancy rate.

It is easy to become enthusiastic about any treatment of male infertility that is performed without adequate controls, even crypt-azoospermia. We all have seen men who are initially azoospermic, who will eventually, in subsequent semen analyses, have spermatozoa in the ejaculate without any treatment [18]. In fact in the early days of ICSI, this was commonly called “crypt-azoospermia” [1, 30]. Without a control group to compare with, one should not be surprised to see a spontaneous pregnancy rate of 9–23% without any treatment of the male partner with severe sperm defects, particularly if the couple has had a short period of infertility and/or if the wife is young [30, 31].

MacLeod and Gold, as far back as 1951 [39], first demonstrated that sperm concentration and motility tend to increase with repeated testing in oligozoospermic and asthenozoospermic men despite no treatment. This was a peculiar mathematical quirk related to the highly variable nature of the sperm count. This means that, without any treatment whatsoever, if you continue to get sperm counts and semen analyses longitudinally on men who initially have low sperm counts and poor motility, the low sperm count and the poor motility will routinely tend to increase with repeated tests and no treatment. Baker et al. were the first to clearly and mathematically explain this phenomenon of “regression toward the mean” [15, 18, 20]. “Regression toward the mean” has profound implications for all clinical trials. Whenever there is a highly variable measurement, if patients have a controlled period followed by a treatment period, there is likely to be a significant improvement even if the treatment is ineffective. Baker et al. observed the same phenomenon that McLeod and Gold had observed 30 years earlier that sperm concentration and motility increased progressively in their study of day-to-day variability of semen analyses in infertile men [38]. Sperm motility increased equally on both active drug and on placebo treatment in a double-blind controlled trial of erythromycin for asthenozoospermia [16]. Clearly, erythromycin had no impact whatsoever on either sperm count or sperm motility. However, in this double-blind control study, it was obvious that the sperm motility increased in an equal manner in patients that were on erythromycin and patients that were on placebo. “In a similar fashion, sperm motility increased in men with varicoceles whether or not they had testicular vein ligations performed” [12]. No matter what the treatment, whether erythromycin, or watchful waiting, clomiphene citrate, or varicocelectomy, an initially low sperm count (because of intrinsic variability) will gravitate higher because of “regression toward the mean.”

Baker and Kovacs also concluded in 1985 that although “a group of subjects selected for low results will on average have higher results on re-measurement,” conversely, a group of men with high sperm counts will on average have lower results after any treatment [18]. Repeated tests after treatment of oligospermia will generally show an increase which has nothing to do with biology but is simply a mathematical event that has to occur. As Baker and Kovacs showed, therefore, a low sperm count will generally improve, with or without any treatment. Similarly, a very high sperm count will generally become lower with or without any treatment. Thus, whenever uncontrolled varicocelectomy studies mention an improvement in motility, or sperm count, this is what one often would expect to find with no treatment whatsoever when you are beginning with oligozoospermic couples [18, 19].

One might, without proper control studies, be very enthusiastic about varicocelectomy in a practice involving younger couples, and less enthusiastic in a practice involving older ones. We discovered a similar confounding phenomenon in the treatment of obstructive azoospermia with sperm retrieval and ICSI. The only factor that significantly affected the variation in pregnancy rate in couples undergoing ICSI with retrieved spermatozoa was the age of the wife [38, 40]. Thus, it seems that in any kind of infertility treatment for male factor, regardless of sperm count, and whether for varicocele or obstructive azoospermia, the most important confounding factor, aside from duration of infertility, is the age and ovarian reserve of the wife.

As long ago as 1978, Rodriguez-Rigau et al. reported a large group of patients which was not prospective and not randomized, but was controlled, some of whom underwent varicocelectomy and others who did not [41]. Rodriguez-Rigau et al. noted a slightly increased percentage motility in patients undergoing varicocelectomy. However, there was no difference in pregnancy rate among those who had varicocelectomy versus those who did not. Furthermore, there was no relation of improvement in post-operative sperm count to pregnancy rate. Those patients who conceived after varicocelectomy had a mean sperm count of 28 × 10 6 spermatozoa/ml and those who did not conceive had a mean sperm count of 26 × 10 6 spermatozoa/ml. Of patients with sperm counts of > 10 × 10 6 spermatozoa/ml those who conceived had a mean sperm count of 40 × 10 6 spermatozoa/ml and those who did not conceive had a mean sperm count of 48 × 10 6 spermatozoa/ml.

As far back as 1968, Uehling studied the fertility of 440 married men in the military coming in for routine physical examination, with and without varicoceles. Of this group, 138 had no children (31.4%) and 302 did have children (68.6%). To break it down further, of the 75 men with a varicocele, 69% had children and of the 227 men without a varicocele, 68% had children. Thus, there was no difference in fatherhood of those young married military recruits who had varicocele versus those who did not have varicocele. The presence or absence of a varicocele in these young men had no influence on whether or not their wives were able to get pregnant [23]. At least in young men, varicocele seemed to have no negative impact on fertility.

So what is the prevalence of varicocele in a group of otherwise healthy young men? Thomason et al. in a similar study of military recruits, in 1979, concluded, “It is apparent that the prevalence of varicoceles in young men occurs with significant frequency and does not interfere with the fertility in all individuals” [24]. It was found that 30.7% of all recruits had a left varicocele (14% were small, and 16.7% were moderate or large), and 29.4% of recruits who had fathered children also had a varicocele (15% were moderate or large). This is similar to the frequency of large or moderate left varicocele in older vasectomy reversal patients [21]. They concluded, “the prevalence of a left-sided varicocele occurs with such frequency among a group of healthy men that one would question the association of a varicocele and poor semen quality.” Furthermore, we have observed no difference in fertility after vasovasostomy in older men with or without varicocele [21]. Nonetheless many adults with varicocele do have atroplued left testicle causing some concern that the varicocele could be the cause.

It is therefore likely that there will be a widespread resurgence in varicocelectomy because of all the newer studies that are contradicting previous negative results. However it is by no means clear that such a resurgence is justified. 
